# A comparative analysis to depict underlying attributes that might determine successful implementation of local adaptation plans

**DOI:** 10.1016/j.envsci.2020.12.002

**Published:** 2021-03

**Authors:** Silvia Rivas, Yeray Hernandez, Ruben Urraca, Paulo Barbosa

**Affiliations:** a European Commission, Joint Research Centre, Via Fermi 2749, I-21027, Ispra, Italy; b Department of Mechanical Engineering, University of La Rioja, 26004, Logroño (La Rioja), Spain

**Keywords:** Climate change, Adaptation, Covenant of Mayors, Stakeholder and citizen participation

## Abstract

Current trends in climate change indicate that the impact on the most vulnerable systems will increase. Urban areas, which concentrate population, economic activity and infrastructures, are sometimes at high-risk locations. Yet they are to be considered as vulnerable systems in need of harmonized structures supporting their efforts towards mitigating climate effects and/or adapting their territories to them. One current structure is the Covenant of Mayors for Climate and Energy (CoM) initiative, tackling in a global and harmonized way local adaptation to climate change. Do CoM cities that developed acceptable climate change adaptation plans have similar characteristics? It is still unclear which might be the drivers or key attributes potentially leading to successful planning within the initiative. In this paper, we explore attributes of the first 51 cities that have submitted their adaptation plans to CoM, in order to identify common elements among accepted plans. Therefore, our hypothesis is that there must be attributes determining the acceptance of adaptation plans. In order to do so, the cities were classified as compliant and non-compliant with the CoM principles. Fisher’s and Kolmogorov-Smirnov tests were applied to identify attributes that are statistically different between both groups. Results show that the engagement of multiple stakeholders and citizens, particularly at the local level, might significantly facilitate the acceptance of adaptation plans in the initiative. We also found that the benefits of stakeholder and citizen engagement could be greater in small municipalities because citizens and stakeholders have more opportunities to participate.

## Highlights

• First analysis to depict key attributes driving successful local adaptation planning.
• Stakeholder engagement as main attribute leading to effective adaptation planning.
• Small population may improve stakeholder engagement and thus the adaptation plans.

## 1 Introduction

Climate change is occurring and poses risks for human and natural systems. The high frequency of heat waves, heavy precipitations, floods, and droughts is already impacting the most vulnerable systems (IPCC, 2014a, 2014b) and the current studies show that increasing effects are expected (Aguiar et al., 2018; Madsen et al., 2014). Urban areas are usually concentrating population, economic activity, and infrastructures in high-risk locations; therefore, taking an increasingly active role in climate policy action is needed (Campos et al., 2017). Cities are in need of harmonized structures supporting or guiding their efforts towards mitigating climate effects and or adapting their territories to them (Araos et al., 2016), like the Covenant of Mayors initiative (EU MEX/15/5840 IP/16/2247).

The Covenant of Mayors for Climate and Energy (CoM) initiative, launched back in 2015 by the European Commission, is a climate change action mainstreamed at local government level tackling adaptation to climate change. This local initiative is based on three pillars: mitigation, adaptation to climate change and secure, affordable and sustainable access to energy. Yet, local authorities engage in climate action with the commitment of producing a feasible and measurable Sustainable Energy and Action Plan (SECAP) on their territories. Particularly, in this paper, we focus on the adaptation strategies that have already been submitted to CoM.

The adaptation to climate change strategy of SECAPs needs to be based on a robust climate risk assessment (Neves et al., 2016). In this assessment, local authorities select the main risks affecting their territories and the most affected sectors of activity by risk identified. These outputs are the base for developing an adaptation strategy, composed of concrete actions tackling selected risks impacting specific sectors of activity. The European Commission’s Joint Research Centre (JRC) is the scientific body of the initiative, developing guidance materials (Bertoldi, 2018a, 2018b, 2018c), adapting methodologies in all regions of the world (Rivas et al., 2018), as well as assessing the overall initiative (Bertoldi et al., 2020) and ensuring the robustness of the submitted plans by providing guidance and feedback to signatories.

There have been several global-scale analyses of adaptation strategies at the local level. Most studies have focused on identifying drivers in the development of local adaptation plans (Araos et al., 2016; Reckien et al., 2018, 2015) by comparing cities with and without local adaptation planning. Araos et al. (2016) evaluated the degree of development of adaptation measures, whereas Reckien et al. (2018) explored the spatial dimension of the adaptation plans. In this line, Kim and Grafakos (2019) also analysed the level of integration of adaptation and mitigation policies in 44 Latin American cities. Other studies have analysed the adaptation strategies by focusing on the indicators and metrics (Arnott et al., 2016), the resilience and adaptive capacity of the cities (Filho et al., 2019; Woodruff et al., 2018), or by comparing resilience plans with the local adaptation plans.

In this study, we focus on the adaptation plans within a specific harmonized framework, the Covenant of Mayors. Compared to previous works, which are mostly focused on large cities, this study presents an insight from a framework designed for cities of all sizes. In fact, 90 % of the current CoM cities are local authorities under 50.000 inhabitants (Bertoldi et al., 2020). The paper is particularly focused on identifying common attributes leading to the acceptance of adaptation plans in the CoM initiative. Our goal is to help cities around the world aiming at joining the initiative to develop their plans, in order to facilitate their access to CoM technical support and financing tools. To do so, we classified the first 51 adaptation plans submitted into complaint and non-complaint with the CoM evaluation criteria, and then we conducted a comparative analysis between both groups searching for potential drivers of the acceptance of the plans. The list of potential drivers is extracted from the CoM reporting framework. The 51 municipalities used are homogeneously distributed among European countries, covering a wide range of population size, from small-sized towns to large cities.

In Section 2, a literature review of comparative analysis is carried out. In section 3, the materials and methods used are presented. Results are presented in Section 4 and discussed in Section 5. Lastly, Section 6 concludes with final remarks.

## 2 Comparative analysis in climate change adaptation

Comparative analyses are considered useful tools commonly used by the climate change community to confront different scenarios, situations or actions. In general, comparative analyses can be applied to either quantitative or qualitative information, or both when necessary. A quantitative case approach to be mentioned is the comparison of two surveys conducted with two years of difference in Australia, in order to detect changes in people’s opinion with regards to the deployment of renewable energies and nuclear power after the nuclear disaster of Fukushima (Bird et al., 2014). Holt et al. (2016) compared the potential impacts of climate change on the primary production of regional seas using projections for the end of the 21^st^ century.

Qualitative comparative analyses can also be found in the literature. Gasper et al. (2013) compared the Human Development Report 2007/8 and the World Development Report 2010 using a combination of frame and content analysis, focusing on lexical choice (by word counting) so as to depict different worldviews. A similar approach was applied to compare the amount of media attention to climate change in different countries with diverse vulnerabilities (Schmidt et al., 2013). European Union cooperation projects have also been compared by means of fuzzy-set analysis to determine to what extent certain conditions may have an impact on learning outcomes (Vinke-de-Kruijf et al., 2020). A fuzzy-set qualitative comparative analysis has also been applied to illustrate how power is distributed, level of coordination, and adaptive capacity in water governance (Pahl-Wostl and Knieper, 2014). A relevant qualitative comparative analysis was run on five developing countries so that their position on international climate negotiations could be described (Rong, 2010).

Comparative analyses have also been adopted to analyse climate change adaptation in different fields. Lobaccaro and Acero (2015) developed a comparative analysis to evaluate how vegetation in urban areas may improve the thermal comfort, enhancing the adaptability to extreme heat events. Geographic Information Systems were applied to extract variables related to the ability of urban green spaces to promote adaptation to climate change and urban regeneration (Garcia Sanchez et al., 2018). Li et al. (2020) explored the differences and similarities of United Kingdom and China’s green infrastructure actions to tackle urban flood risks. Witt et al. (2015) compared three alternative methods to evaluate the cost-effectiveness of retrofitting buildings, useful for climate adaptation. Milwicz and Paslawski (2017) compared diverse heating systems for single family housing through a life cycle analysis approach to estimate costs, adaptability and environmental impacts. Díaz López et al. (2019) evaluated the suitability of existing methods to assess the sustainability of buildings. Kumar et al. (2020) presented a comparative analysis of building insulation materials, in terms of thermal conditions, hygroscopic, acoustic conditions, resistance to fire, environmental impact and cost, as well as their performance in different climate conditions.

Clark (2017) applied a comparative analysis to investigate the potentiality of unmanned aerial systems to monitor coastal erosion triggered by storm events. Xian et al. (2018) compared the different flood protection measures undertaken in two megacities, such as Shanghai and New York, in order to search for risk factors that include flood hazard, exposure and vulnerability. Lereboullet et al. (2013) analysed meteorological data, semi-structured interviews, and field observations to compare two viticulture systems’ resilience to climate change. A multi-method approach that integrated surveys, interviews, videos, literature and fieldwork to compare climate change perceptions, policies and knowledge of diverse rural areas can be seen in Smith et al. (2014). Improved water storage and sustainable water use for agriculture in a context of climate change has also been subject of comparative analysis (Baffaut et al., 2020).

Comparative analyses have also been applied to describe how climate change adaptation is mainstreamed into civil protection policies (Groven et al., 2012). More recent work presents the use of indicators to compare the relevance, effectiveness, efficiency, results and impact, sustainability and management of adaptation strategies in Europe (Rutherford et al., 2020).

## 3 Materials and methods

### 3.1 Adaptation plans within the Covenant of Mayors

**Fig. 1:**
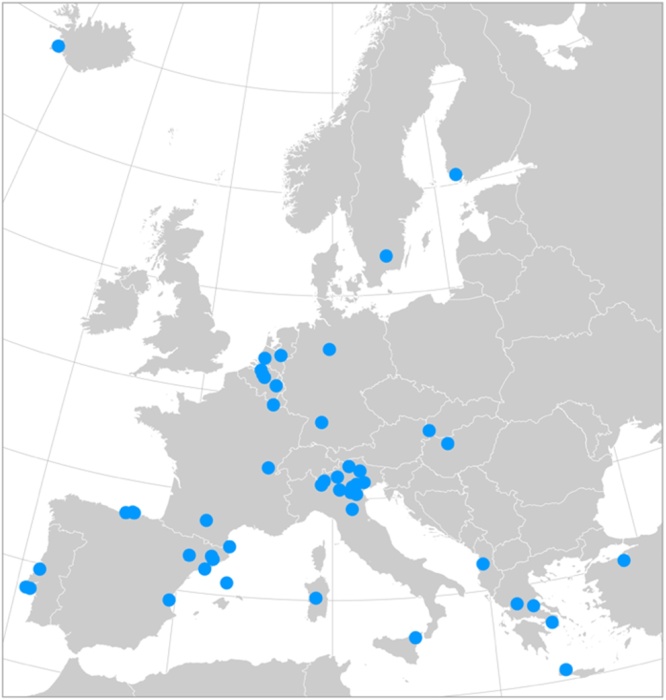
Covenant of Mayors’ municipalities used in the study.

In November 2018, a total of 51 European local authorities completed the submission of their Risk and Vulnerabilities Assessment (RVAs) as well as the set of actions to adapt to the expected risks, the so-called “adaptation plan”. European municipalities submitted their plans to MyCovenant platform based on an excel-based reporting tool, now on-line, developed jointly by the Covenant of Mayors Office (CoMOffice) and the JRC. Cities in Europe could commit to adaptation goals in the frame of the CoM only after 2016 and they had two years to complete their adaptation action plans. This is the reason behind the low representation of adaptation plans in the initiative so far. For this study, the whole set of 51 municipalities, which are evenly distributed across Europe (Fig. 1), was included.

JRC has the mandate of conducting an evaluation of the SECAP submitted by the cities. This evaluation is based on a set of evaluation criteria proposed by Barbosa et al. (2018) that contributes to guaranteeing the credibility and reliability of the whole CoM initiative. The purpose of the evaluation is to ensure that the city is fully compliant with the mandatory criteria of the initiative and therefore is accepted (compliant) or non-accepted (non-compliant). Secondly, recommendations for potential improvements are formulated to the city. The evaluation criteria are divided in five sub-components (Table 1): compliance with the time frame, completeness, coherence, quantification, and progress. These criteria are considered as the minimum requirements such that an adaptation plan can be accepted by the CoM initiative.

**Table 1:** Evaluation criteria within the JRC framework.

Criteria	Key elements
Compliance with the reporting timeframe	RVAs and adaptation action plan
Completeness	Adaptation goals
Internal coherence	Alignment of goals, risks and actions
Quantification	RVAs and adaptation actions
Progress	Adaptation actions

*Source:*Barbosa et al. (2018).

The JRC adaptation team evaluated the 51 adaptation plans based on the previous evaluation criteria. As a result, the local action plans were classified in terms of performance within the initiative. Out of the 51 cities, 21 were classified as compliant with the CoM evaluation criteria, while 30 cities were classified as non-compliant.

### 3.2 Statistical analysis

A statistical comparative analysis between compliant and non-compliant cities was conducted based on different attributes of the adaptation plans. We hypothesized that if an attribute shows a statistical different between both groups, the attribute may be a potential driver in the acceptance of adaptation plans. The statistical analysis was performed using nonparametric tests due to the small sample size and the non-normality of most of the attributes.

The attributes selected were extracted from several sections of MyCovenant reporting tool, reflecting all the elements that are considered as mandatory for the city to report. They were grouped into three main categories (Table 2): administrative attributes, financial resources, and high-level hazards currently threatening the cities. The first group includes administrative characteristics of the city and the city plan development, such as city size, population size, administrative structure within the municipality, level of stakeholder engagement, and level of adaptation commitment represented by the definition of concrete adaptation goals. The second group focuses on the different financing lines the city foresees for the development, implementation and monitoring of the adaptation plan. Finally, the third group shows the hazards described by the cities as potential or current climatic threats.

**Table 2:** Attributes used to conduct the statistical analysis.

Category	Attribute	
Administrative attributes	Population size	
	Population density	
	Stakeholders with high level of participation	Local authority’s staff
		External stakeholders at local level
		Stakeholders at other levels of governance
	Adaptation goals defined	
	Administrative structure coordinating the plan	

Financial resources	Local authority’s own resources	
	National funds	
	EU funds	
	Private sources	

High-level hazards	Droughts	
	Extreme heat	
	Floods	
	Forest fires	
	Sea level rise	
	Landslides	
	Storms	
	Extreme cold	

Note that in this study, both the evaluation criteria (Barbosa et al., 2018) and the list of attributes studied as potential drivers (Covenant of Mayors Office - Europe, 2020) were imposed by the CoM framework. Both are the result of several years of international committees that included a group of practitioners represented by cities of all kind and from all regions of the world. Moreover, when the Covenant of Mayors became global in 2016, a specific committee on adaptation was built with representatives from EU, UN habitat, ICLEI, Climate Alliance, Energy Cities, and C40. Therefore, the evaluation criteria and the reporting framework include the feedback of city representatives and adaptation planners gathered throughout different consultation processes.

Most of the attributes were categorical values with a binary response, resulting in 2 × 2 contingency tables when comparing the two groups of cities. These attributes were evaluated with Fisher's exact test, a nonparametric test designed to compare the distribution of categorical variables between two groups. The chi-square test is the other test used in the literature for this purpose. However, chi-square test applies an approximation assuming a large sample size, whereas Fisher’s test is exact. Thus, Fisher’s test is better suited for small samples, and particularly for 2 × 2 contingency tables with low frequencies such as the ones of the current study (Kim, 2017). Note that Fisher’s test is valid for any sample size and it steadily converges with chi-square test when the sample size increases. The null hypothesis H_0_ is that there is no difference between the distributions of the two groups. Therefore, if H_0_ is rejected, we can state that there is a significant difference in the evaluated attribute between the two groups.

Some of the categorical attributes can be grouped in one of the following categories: stakeholder engagement, financial resources, and hazards. In these cases, Fisher’s test was also calculated for the accumulated values, e.g., the total number of hazards identified by a city. Combining different categories is recommended when having low frequencies to increase the identification of significant relationships (Allam et al., 2017). Note that these three categories cannot be treated as three categorical variables because the cities can report more than one attribute within each category, e.g., a city can identify several types of hazards.

There are also two attributes that are not strictly categorical variables: defining adaptation goals, and the three types of stakeholders. In these cases, cities can have more than one attribute of the same class, e.g., a city can define five adaptation goals or report the participation of six local stakeholders. These attributes could have been analysed as quantitative variables (e.g., number of adaptation goals per city) if a sufficiently large sample size was available. However, due to the small sample size, they were analysed as two categorical scenarios to enlarge significant relationships: (i) cities with at least one adaptation goal or one stakeholder engaged, and (ii) cities with more than one adaptation goal or more than one stakeholder engaged.

The two quantitative attributes, population size and density, were evaluated with the two-sample Kolmogorov-Smirnov (KS) test. The KS is a nonparametric test that makes no assumption about the distribution of the data. It evaluates whether two samples come from the same distribution by comparing their cumulative distribution functions (CDFs). The *p-value* is calculated based on the maximum distance between both CDFs. The null hypothesis H_0_ is that both groups were drawn from the same distribution. Therefore, if H_0_ is rejected, we can state that the distribution of the evaluated attribute is significantly different between the two groups.

A significance level of 0.05 was used, rejecting the null hypothesis if *p-value* < 0.05. Nonetheless, it should be noted that statistical tests are sensitive to sample size. As sample size increases, absolute differences become a smaller and smaller proportion of the expected value. What this means is that a reasonably strong association may not come up as significant if the sample size is small, and conversely, in large samples, we may find statistical significance when the findings are small and uninteresting, i.e., the findings are not substantively significant, although they are statistically significant. Consequently, the results obtained will also be discussed and confronted with the existing literature on the matter.

Note that the results of the statistical tests do not allow concluding anything more concrete that there is some link in the sample between the attributes and the performance of adaptation plans (Voelker et al., 2001). It does not necessarily imply that one variable has any causal effect on the other.

## 4 Results

### 4.1 Administrative attributes

**Fig. 2:**
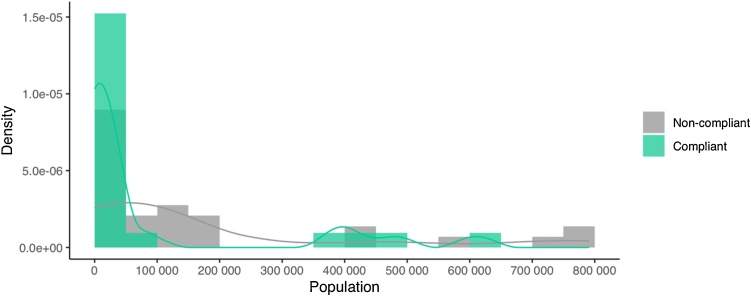
Histogram of population size with a density curve calculated with a cosine kernel function. The statistical significance between both groups was calculated with the two-sided KS test (***p-value* = 0.020**).

**Fig. 3:**
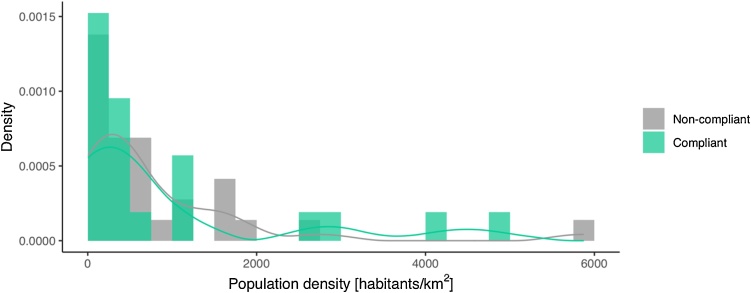
Histogram of population density with a density curve calculated with a cosine kernel function. The statistical significance between both groups was calculated with the two-sided KS test (*p-value* = 0.979).

The two-sided KS test was used to (Fig. 2). Results show that that there is a significant difference between the population size of compliant and non-compliant cities (*p* = 0.020). The number of small municipalities (population < 50,000) in the compliant group almost doubles that in the non-compliant one (Fig. 2). The 76.2 % of compliant municipalities have less than 50 000 habitants, while this value decreases to a 43.3 % for non-compliant ones. On the contrary, the population density distributions (Fig. 3) of both groups are statistically similar (*p* = 0.979).

**Fig. 4:**
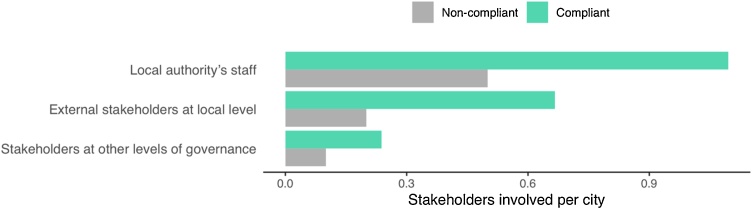
Number of stakeholders with a high level of participation engaged per city.

The influence of the number of stakeholders involved in the development of adaptation plans was studied with two comparative analyses. Table 3 shows the statistical significance of having at least one stakeholder engaged in the different categories defined. In this case, neither the individual types of stakeholders nor the cumulative sum showed a link with the acceptance of adaptation plans. However, the results changed when analysing the participation of multiple stakeholders (Table 4). In this case, a significant difference (*p* = 0.009) was observed between compliant and non-compliant cities in terms of the participation of local authority’s staff, i.e., all the Departments of the Municipality involved in climate change-related matters. Besides, the cumulative sum also shows that overall, extending the participation to multiple stakeholders has a link with the acceptance of adaptation plans (*p* < 0.001). This fact was corroborated with the analysis exact number of stakeholders participating per city (Fig. 4). The bar plots show that for all types of stakeholders, the number of stakeholders engaged per city in compliant cities doubles that of non-compliant ones.

**Table 3:** Cities with at least one stakeholder engaged at high level of participation. The percentage of cities per group is shown in brackets. *p-values* were calculated with Fisher’s exact test. Bold values indicate *p* < 0.05.

Attribute	Compliant	Non-compliant	*p-value*
Local authority’s staff	15 (71 %)	15 (50 %)	0.156
External stakeholders at local level	9 (43 %)	6 (20 %)	0.119
Stakeholders at other levels of governance	3 (14 %)	3 (10 %)	0.680

Total	27 (43 %)	24 (27 %)	0.055

**Table 4:** Cities with multiple stakeholders engaged at high level of participation. The percentage of cities per group is shown in brackets. *p-values* were calculated with Fisher’s exact test. Bold values indicate *p* < 0.05.

Attribute	Compliant	Non-compliant	*p-value*
Local authority’s staff	5 (24 %)	0 (0%)	**0.009**
External stakeholders at local level	2 (10 %)	0 (0%)	0.165
Stakeholders at other levels of governance	1 (5%)	0 (0%)	0.412

Total	8 (13 %)	0 (0%)	**<0.001**

Concerning the number of goals defined in the adaptation plans (Table 5), neither defining adaptation goals (*p* = 0.978) nor defining multiple adaptation goals (*p* = 0.744) were found to have a statistical link with the acceptance of adaptation plans. Besides, neither the existence of an administrative structure in charge of coordinating the adaptation plan (Table 6) had a link with the acceptance of the plans (*p* = 0.083).

**Table 5:** Number of cities with adaptation goals defined. The percentage of cities in each group is shown in brackets. *p-values* were calculated with Fisher’s exact test.

Attribute	Compliant	Non-compliant	*p-value*
Adaptation goals defined	6 (29 %)	8 (27 %)	0.978
Multiple adaptation goals defined	5 (24 %)	6 (20 %)	0.744

**Table 6:** Number of cities having an administrative structure coordinating the plan. The percentage of cities per group is shown in brackets. *p-values* were calculated with Fisher’s exact test. Bold values indicate *p* < 0.05.

Attribute	Compliant	Non-compliant	*p-value*
Administrative structure coordinating the plan	9 (43 %)	21 (70 %)	0.083

### 4.2 Financial sources

**Fig. 5:**
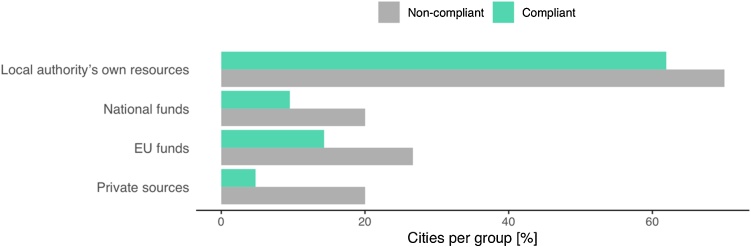
Number of cities using each type of financial resource.

Financial resources used by CoM signatories are classified in four main categories (Table 7 and Fig. 5). Cities can use different types of financial resources, so each category was statically analysed individually. According to the test carried out for our sample, the acceptance of the plans was statistically independent of the availability of local funds (*p* = 0.563), national funds (*p* = 0.445), EU funds (*p* = 0.490), and private funds (*p* = 0.217). These two variables are also independent when analysing the financial resources altogether (*p* = 0.087).

**Table 7:** Financial resources used by each city. The percentage of cities per group is shown in brackets. *p-values* were calculated with Fisher’s exact test. Bold values indicate *p* < 0.05.

Attribute	Compliant	Non-compliant	*p-value*
Local authority’s own resources	13 (62 %)	21 (70 %)	0.563
National funds	2 (10 %)	6 (20 %)	0.445
EU funds	3 (14 %)	8 (27 %)	0.490
Private sources	1 (5%)	6 (20 %)	0.217

Total	19 (23 %)	41 (34 %)	0.087

### 4.3 Exposure to climate hazards

**Fig. 6:**
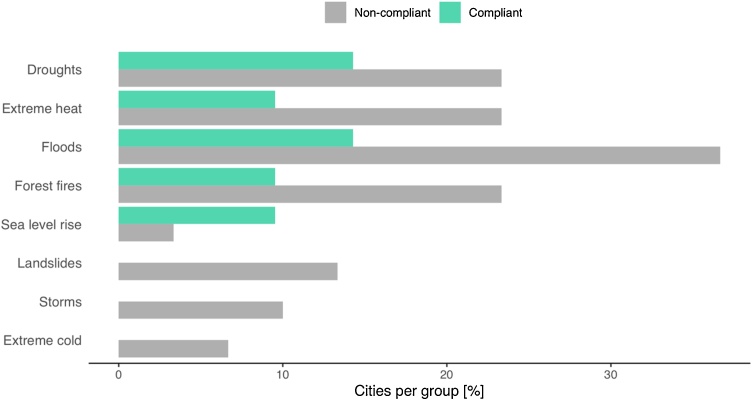
Number of cities reporting each type of hazard (current level high).

The number and type of climate hazards vary with the location of the municipality as well as with the degree of completeness of the adaptation plan. The number of individual hazards was statistically similar between compliant and non-compliant cities (Table 8): droughts (*p* = 0.495), extreme heat (*p* = 0.277), floods (*p* = 0.113), forest fires (*p* = 0.277), sea level rise (*p* = 0.561), landslides (*p* = 0.134), storms (*p* = 0.259), and extreme cold (*p* = 0.506). However, the results changed when analysing the aggregated values. The total number of hazards defined by compliant and non-compliant groups are statistically different (*p* = 0.003). Fig.6 shows that for this subset of municipalities, the number of hazards identified by non-compliant cities is slightly larger than that for compliant ones.

**Table 8:** High-level hazards identified by each city. The percentage of cities per group is shown in brackets. *p-values* were calculated with Fisher’s exact test. Bold values indicate *p* < 0.05.

Attribute	Compliant	Non-compliant	*p-value*
Droughts	3 (14 %)	7 (23 %)	0.495
Extreme heat	2 (10 %)	7 (23 %)	0.277
Floods	3 (14 %)	11 (37 %)	0.113
Forest fires	2 (10 %)	7 (23 %)	0.277
Sea level rise	2 (10 %)	1 (3%)	0.561
Landslides	0 (0%)	4 (13 %)	0.134
Storms	0 (0%)	3 (10 %)	0.259
Extreme cold	0 (0%)	2 (7%)	0.506

Total	14 (7%)	57 (21 %)	**0.003**

## 5 Discussion

The involvement of stakeholders and citizens with a high level of participation was the attribute showing the strongest statistical significance. A link was found between the engagement of multiple stakeholders, particularly at the local level, and the acceptance of adaptation plans (Table 4). Besides, for all types of stakeholders, the number of engagements in compliant cities doubles that of non-compliant ones (Fig. 4). These results might indicate that a higher participation of citizens and stakeholders, particularly at the local level significantly facilitates the acceptance of adaptation plans. Literature available on stakeholder and citizen engagement in climate adaptation supports these results.

Stakeholders are generally engaged in the development of local adaptation strategies (Aguiar et al., 2018). According to Christoforidis et al. (2013) “*the success of possible adopted measures by the local governments is heavily based on the public acceptance and the citizens’ active participation.”* It is said that environmentally sustainable cities are likely to have engaged citizens in their environmental plans (Elelman and Feldman, 2018).

Public participation is believed to be useful for adaptation planning due to several reasons. For example, it has been alleged to be meaningful to evaluate the *statu quo* of adaptation planning and the understanding of existing policies and barriers (Hernandez et al., 2018a). Consequently, public participation prepares the conditions for long term climate goals formulation and visions (Hernandez et al., 2018b; Hilden et al., 2017; Mendizabal et al., 2018). Similarly, citizen participation is said to reinforce the government’s ability to attain the goals established (Larsen et al., 2011).

Stakeholder and citizen engagement also facilitates a common understanding of climate risks, establishing a platform to share knowledge on how to better adapt the risks foreseen (Mendizabal et al., 2018). Furthermore, public participation reinforces deliberation with regards to desirable futures aiming at sustainability (Larsen et al., 2011), stimulating transitions towards more climate-resilient cities (Mendizabal et al., 2018). Public participation also provides a more holistic framework to design (Allam et al., 2017) climate change adaptation actions (Endo et al., 2017; Hernandez et al., 2018a).

The total number of hazards identified per city and population size was also correlated with the acceptance of adaptation plans. However, and compared to stakeholder engagement, these attributes cannot be strictly considered potential drivers for the acceptability of adaptation plans because they are intrinsic characteristics of the municipalities that cannot be modified.

The total number of hazards reported by non-compliant cities was statistically larger than that reported by compliant ones (Table 8), which is in agreement with existing literature. Reckien et al. (2015) found that cities at risk of severe climate change impacts and with a high degree of future vulnerability have fewer adaptation plans. Particularly, they found that cities in low-lying coastal areas and hot climates do not engage more in climate planning, but on the contrary, they engage less. A survey conducted among Norwegian municipalities confirmed that adaptation efforts are driven by past extreme events and not by projected future hazards (Amundsen et al., 2010). Moreover, Reckien et al. (2015) hypothesised that the existence of multiple hazards could be even a barrier due to the financial resources and the infrastructure required for adapting to these hazards. In this line, our results might corroborate that the existence of multiple hazards could be negatively correlated with an effective adaptation planning.

Concerning population size, the population of compliant cities was statistically smaller than that of non-compliant ones (Fig. 2). Literature available about this relationship shows contradictory conclusions. Reckien et al. (2015) and Reckien et al. (2018) analysed the presence of adaptation plans in 200 and 885 medium and large-sized European cities, respectively, finding that large cities are more likely to have adaptation plans than small ones. This could be because small cities have fewer resources and are less likely to be engaged in national and international networks (Lioubimtseva and da Cunha, 2020). On the other hand, other studies suggested that there is not a statistical relationship between population size and the adaptive capacity of large cities (Filho et al., 2019; Kim and Grafakos, 2019). These results contrast with our analysis, but it should be noted that neither the dependent variable nor the characteristics of the cities are comparable with our study. For instance, some of these studies evaluate the presence of adaptation plans while ours focuses on the quality of those adaptation cities. Besides, literature studies are mostly focused on large cities while our subset is composed of the first 51 cities submitting their adaptation plans to CoM. Thus, all municipalities have an adaptation plan, belong to an international climatic network, and have a substantially smaller population than the abovementioned studies. All these differences hinder the comparison of our results with those in existing literature. Nonetheless, if a relationship exists between population size and an acceptable adaptation strategy, this relationship may be weak and strongly dependent on the characteristics of the cities analysed.

A potential link between population size and stakeholder engagement might explain the smaller population of compliant cities. Dahl and Tufte (1973) already pointed out that citizens have more stimulus to participate in decision-making when states are smaller. Similarly, Gilbert et al. (1974) observed a moderate positive correlation between community size and the magnitude of citizen influence in local decision making. However, the opposite was highlighted by Newton (1982) who stated that the democratic advantages of small units of government had often been overestimated whereas their democratic disadvantages concealed. Furthermore, he said that larger units of government are not necessarily more democratically deficient, instead, they could be even more democratic. In this latter current of thought, Martins (1995) indicated that amalgamating local small authorities is positive to guarantee a growing citizens’ familiarity and interest in local public affairs, concluding that small municipalities are not linked to higher levels of citizen participation. However, Frandsen (2002); Larsen (2002) and Rodrigues and Tavares (2020) have recently reached the opposite conclusion, i.e., the amalgamation of small municipalities has resulted in lower participation in the elections. A possible explanation is that people in larger cities are much less inclined to contact officials, attend to community or organizational meetings, or vote in local elections, i.e., generally speaking, people in big cities are less interested in local affairs (Oliver, 2000).

Larsen (2002) also detected that, even though participation is higher in smaller units of government, municipal size does neither influence citizens’ interest in and knowledge of their local politics, nor their perception of local politicians and their trust in local political decisions. In fact, according to Guerring and Zarecki (2014), smaller municipalities may compromise the electoral and liberal dimension of democracy. Besides, they suggested that larger population enhances greater democracy, understood here as voting regularly. Their explanation to this is that larger populations have a more developed check-and-balance system, a more developed capacity to contain conflicts, a more developed political infrastructure, and a higher degree of political institutionalization. More recently, it has been said that jurisdiction size has a causal and sizeable detrimental effect on citizens' internal political efficacy (Lassen and Serritzlew, 2011), maybe because citizens who live in smaller municipalities feel a greater sense of political efficacy and participate more in local politics (McDonnell, 2020).

Even though there has been a long discussion on this topic, where both sides have indicated their pros and cons, the possible link between smaller population size and citizen and stakeholder engagement seems to have a rationale behind that might be summarised as a larger sense of closeness. Therefore, we believe that the higher acceptance rate of adaptation plans in small municipalities may be partly explained by the greater ability for stakeholder and citizen participation.

## 6 Conclusions

In this period of developing adaptation strategies in the framework of the Covenant of Mayors initiative, it was unclear which might be the drivers or key attributes that could potentially lead to developing acceptable adaptation plans. In order to identify those potential drivers, we have analysed the first 51 municipalities that have submitted their adaptation plans to the Covenant of Mayors. This analysis was conducted aiming at having a first clue of attributes that might explain the acceptability of the adaptation plans.

The limitations of the study, due to the few plans submitted at the moment, do not allow us to have a thorough understanding to what extent other attributes could play a role in the potential success of the Energy and Climate Action Plans. Actually, we do not deny the importance of all the attributes analysed here, such as having an administrative structure, clear adaptation goals, or allocated funding. Until further analysis is developed, we could only state that the engagement of multiple stakeholders and citizens might drive to effective adaptation planning. We also observed that the benefits of stakeholder and citizen engagement could be greater in small municipalities because participatory processes can be developed more easily. Our findings could be relevant for the more than 9 000 cities (10 % of world population) that are nowadays developing adaptation strategies under the Covenant of Mayors framework. Helping them in joining the initiative could give them harmonized tools to develop successful local action adaptation strategies. Furthermore, joining could lead them to engage in international processes of technical and financial support that would facilitate the actual implementation of the plans.

## CRediT authorship contribution statement

**Silvia Rivas:** Conceptualization, Methodology, Investigation, Writing - original draft. **Yeray Hernandez:** Conceptualization, Methodology, Investigation, Writing - original draft. **Ruben Urraca:** Formal analysis, Writing - original draft, Visualization. **Paulo Barbosa:** Writing - review & editing.

## Declaration of Competing Interest

The authors report no declarations of interest.
